# Evaluating the genotoxicity of salinity stress and secondary products gene manipulation in lime, *Citrus aurantifolia*, plants

**DOI:** 10.3389/fpls.2023.1211595

**Published:** 2023-07-12

**Authors:** Hadeer Darwish, Ghaida S. Al-Osaimi, Najla Amin T. Al Kashgry, Hana Sonbol, Aisha A. M. Alayafi, Nadiyah M. Alabdallah, Abdulrahman Al-Humaid, Nadi Awad Al-Harbi, Salem Mesfir Al-Qahtani, Zahid Khorshid Abbas, Doaa Bahaa Eldin Darwish, Mohamed F. M. Ibrahim, Ahmed Noureldeen

**Affiliations:** ^1^Department of Biotechnology, College of Science, Taif University, Taif, Saudi Arabia; ^2^Department of Medicinal and Aromatic Plants, Horticulture Research Institute, Agricultural Research Center, Giza, Egypt; ^3^Department of Biology, College of Science, Taif University, Taif, Saudi Arabia; ^4^Department of Biology, College of Science, Princess Nourah Bint Abdulrahman University, Riyadh, Saudi Arabia; ^5^Department of Biological Sciences, Faculty of Science, University of Jeddah, Jeddah, Saudi Arabia; ^6^Department of Biology, College of Science, Imam Abdulrahman Bin Faisal University, Dammam, Saudi Arabia; ^7^Basic and Applied Scientific Research Center, Imam Abdulrahman Bin Faisal University, Dammam, Saudi Arabia; ^8^Plant Production and Protection Department, College of Agriculture and Veterinary Medicine, Qassim University, Buraydah, Saudi Arabia; ^9^Biology Department, University College of Tayma, University of Tabuk, Tabuk, Saudi Arabia; ^10^Department of Biology, Faculty of Sciences, University of Tabuk, Tabuk, Saudi Arabia; ^11^Botany Department, Faculty of Science, Mansoura University, Mansoura, Egypt; ^12^Department of Agricultural Botany, Faculty of Agriculture, Ain Shams University, Cairo, Egypt; ^13^Department of Agricultural Zoology, Faculty of Agriculture, Mansoura University, Mansoura, Egypt

**Keywords:** *Citrus aurantifolia*, salinity stress, comet assay, terpenes, transcriptome

## Abstract

Salinity is a significant abiotic stress that has a profound effect on growth, the content of secondary products, and the genotoxicity of cells. Lime, *Citrus aurantifolia*, is a popular plant belonging to the family Rutaceae. The interest in cultivating this plant is due to the importance of its volatile oil, which is included in many pharmaceutical industries, but *C. aurantifolia* plants are affected by the NaCl salinity levels. In the present study, a comet assay test has been applied to evaluate the genotoxic impact of salinity at 0, 50, 100, and 200 mM of NaCl on *C. aurantifolia* tissue-cultured plants. Furthermore, terpene gene expression was investigated using a semi-quantitative real-time polymerase chain reaction. Results from the two analyses revealed that 200 mM of NaCl stress resulted in high levels of severe damage to the *C. aurantifolia* plants’ DNA tail 21.8%, tail length 6.56 µm, and tail moment 3.19 Unit. The relative highest expression of RtHK and TAT genes was 2.08, and 1.693, respectively, when plants were exposed to 200 mM of NaCl, whereas pv4CL2RT expressed 1.50 in plants subjected to 100 mM of NaCl. The accumulation of transcripts for the RTMYB was 0.951 when plants were treated with NaCl at 50 mM, and RtGPPS gene was significantly decreased to 0.446 during saline exposure at 100 mM. We conclude that the comet assay test offers an appropriate tool to detect DNA damage as well as RtHK, TAT, and pv4CL2RT genes having post-transcriptional regulation in *C. aurantifolia* plant cells under salinity stress. Future studies are needed to assess the application of gene expression and comet assay technologies using another set of genes that show vulnerability to different stresses on lime and other plants.

## 1 Introduction

Plant genome persistence is impacted by salinity environmental stress, which also has a significant negative effect on crop productivity and is clearly associated with social, economic, and environmental issues in the affected regions. Globally, soil salinity has a greater impact on agricultural output than other factors, making reclaimed land unusable for farming as the salinity ratio rises (Saira et al., 2014). Researchers have looked into the various stress factors’ effects on plant genotoxicity. It has been demonstrated that the comet test is a promising technique for detecting damage to DNA and its repair capability at the single-cell level, despite that salinity is one of the most important abiotic factors that affect DNA and cause genome damage (Tomas et al., 2000). The use of plant comet assay has significantly expanded in recent years due to its precision, simplicity, and requirement of just one cell for valid results. For many years, only a few plants, such as *Arabidopsis thaliana*, *Allium cepa*, *Vicia faba*, or *Nicotiana tabacum*, were used in comet assays; however, in recent years, the number of these plants has significantly expanded (Gichner et al., 2008; Ventura et al., 2013). In earlier research, comet assay has been used to assess the potential genotoxicity of substances such as heavy metals, radiation, phytocompounds, pesticides, polluted complex matrices, and nanoparticles (Ghosh et al., 2015). The most recent information regarding this technique’s use as an effective method for examining the genotoxic effects of salinity stress conditions on lime (*Citrus aurantifolia*) plants will be reviewed in this paper. The most famous plant family in the world is Rutaceae, and nearly all of its members have volatile oil glands that are highly concentrated in the terpene compound. Medicinal and aromatic crops have a variety of uses and benefits. The member of this family known as the lime, *C. aurantifolia*, is popular and well-liked. The plant has a long history of use in aromatherapy, cosmetics, pharmacy, flavoring, and perfumes in addition to treating sleep problems (Sonwa, 2000; Labra et al., 2004). The lime plant is often a tiny shrub-like tree that grows to a height of approximately 5 m. It is a tree that is both evergreen and everbearing, is thick and erratically branching, and has short, stiff spines (thorns). The lime tree produces fruits and flowers throughout the year, although in the Northern Hemisphere, it blooms most profusely from May to September. The peel of the fruits is incredibly thin, and the pulp vesicles are a yellow-green color. The fruit juice has a high vitamin C concentration and is acidic and aromatic, just as sour as lemon juice, but is more aromatic. *C. aurantifolia* is spiky, weaker, and less robust, and it needs more heat for fruit production. Due to the non-climacteric nature of limes, they mature and ripen on the tree (Mumtaz-Khan et al., 2017).

Through a study program using *in vitro* selected NaCl-tolerant plants, tissue culture techniques have been often exploited as a potential approach to explain the cellular mechanisms involved in salt tolerance. Because growth conditions may be easily managed in plant cell cultures, some studies have avoided growing whole plants by using plant tissue culture instead (Davenport et al., 2003; Gu et al., 2004). The salinity of soils is quickly returning to be a serious issue in some locations where saline water is utilized for irrigation, as well as in reclaimed land. These characteristics prompted the development of new cultivars that could be grown in salinized soil, making it simpler to choose the right cultivar with salt tolerance. Due to the significant amount of various essential oil constituents that are extracted from *C. aurantifolia* plants, these plants have commercial significance. Due to the significant production of a variety of essential oil components from various cultivars, these plants are significant commercially. Terpenoids and derivatives of the phenylpropane ring have been discovered as the main volatile components of *C. aurantifolia* oil (Boussaada and Chemli, 2006; Boussaada et al., 2007). Terpenoids are among the most diverse types of secondary metabolites found in plants. They play a role in a variety of biological processes, such as growth and development, photosynthesis, and respiration (Gershenzon and Kreis, 1999; Rodriguez and Boronat, 2002), along with other biological compounds like hormones, sterols, carotenoids, brassiteroids, and abscisic acid that aid in plant defense against pathogens and herbivores as well as in plant reproduction through pollinator attraction and seed dispersal (Dudareva et al., 2006).

Genome research may reveal details on the mechanism of action and transcriptional behavior of the genes involved in terpene biosynthesis (Ashour et al., 2010). As a result, investigating elective genes that may be connected to this biological target may be possible. The linalool synthase enzyme (RtLS) is significantly associated with linalool accumulation and transcript levels for the linalool synthase gene (Lane et al., 2010). Hexokinase synthase (RtHK), a glycolytic enzyme, is in charge of accelerating the transformation of ATP-dependent hexoses into hexose 6-phosphates (Jyan et al., 1997), similar to how hydroxycinnamate ester and amide pathway genes’ primary enzymes, hydroxycinnamic acid CoA-ligase synthase (Pv4CL2RT), are predominantly controlled at the transcriptional level (Dixon and Paiva, 1995; Holton and Cornish, 1995). Marzieh et al. (2019) confirmed that the synthesis of α-terpinyl acetate, 1,8-cineole, and linalyl acetate, the major constituents of the terpenoids stored in *Salvia mirzayanii* leaves, was induced by salinity stress. However, the increased content of linalyl acetate was completely different from that of another compound. According to this hypothesis, plants change the demand for essential terpenoid concentration as well as composition when responding to incoming environmental stresses. Similarly, Karray-Bouraoui et al. (2009) showed that salt stress enhanced essential oil yield in *Mentha pulegium* by approximately 2.75-fold and affected the percentage of menthone, which is the major compound in that species (∼51%), increasing menthone, pulegone, and neomenthol, the principal components of the monoterpene group. The chemical composition of essential oil in clary sage (*Salvia sclarea* L.) was also strongly affected by NaCl treatments (Taarit and Msaada, 2010). Flavonoids synthase (RTMYB), a crucial enzyme in biosynthesis reactions, regulates the expression of dihydroflavonol reductase genes required for the production of 3-deoxyflavonoids (Boddu et al., 2006). Citrus fruit anthocyanin production genes are activated by the transcription factor RTMYB (Butelli et al., 2012). A monoterpene biosynthesis enzyme known as RtGPPS is found in plastids and is known to be plastid localized (Tholl et al., 2004). In a previous study, Shirazi et al. (2019) evaluated the impact of salinity stress on the expression of key genes including squalene synthase (*SQS1* and *SQS2*), β-amyrin synthase (*bAS*), lupeol synthase (*LUS*), cycloartenol synthase (*CAS*), β-amyrin 11-oxidase (*CYP88D6*), and β-amyrin 24-hydroxylase (*CYP93E6*), which are involved in the biosynthetic pathway of triterpenoids in licorice root. The reversible interconversion of amino acids and their 2-oxoacid cognates is catalyzed by tyrosine aminotransferases, which are widely distributed enzymes. Typically, 2-oxoacid acts as the amino acceptor and an amino acid as the amino donor. It is involved in numerous metabolic processes, including the anabolism and catabolism of amino acids, vitamin biosynthesis, assimilation of carbon and nitrogen, secondary metabolism, gluconeogenesis, and aminotransferases (Liepman and Olsen, 2004).

This study aimed to determine the impact of salinity stress levels on the induction of genetic damage using a comet assay technique and to evaluate the transcriptional level connected with gene expression (terpene-related genes) in *C. aurantifolia* plant tissue cultures.

## 2 Materials and methods

The current study was conducted from January 2022 to December 2022 in the Molecular Biology and Plant Tissue Culture Laboratories at the Faculty of Science, Taif University, Saudi Arabia.

### 2.1 Plant materials and *in vitro* salinity assay

*C. aurantifolia* seeds were obtained from the Al-Shafa region of Taif, Saudi Arabia, sterilized by washing with ethanol 70% containing a few drops of tween 20 for 30 s, rinsed three times with sterilized distilled water, and then soaked for 10 min in 10% commercial Clorox solution (1% sodium hypochlorite) under a sterilized condition on a laminar air flow hood. They were then washed five times with sterile distilled water. Three sterilized seeds per jar were aseptically inoculated on nutritional (Murashige and Skoog, 1962) medium (MS) containing 3% sucrose, and 0.7% agar had been used to solidify it. After bringing the pH of the medium to 5.8, the medium was divided among 30 ml of culture jars and sterilized in an autoclave at 121°C for 20 min. For 10 days, the seed cultures were kept at 25°C ± 2°C in the dark. The seedlings were moved under continuous cool white fluorescent light for 16 h every photoperiod at an intensity of 2,000 lux. Explants that had germinated were then subcultured for 2 weeks in sterile conditions using the same MS medium with various concentrations of NaCl (0, 50, 100, and 200 mM), keeping the aforementioned culture requirements. After being preserved under salt stress for 14 days, growth characteristics and genotoxicity effect were evaluated using the standard method described below.

### 2.2 Genotoxicity assay

A petri plate containing Sörensen buffer (50 mM of sodium phosphate and 0.1 mM of ethylene diamine tetra acetic acid (EDTA), pH 6.8) and 0.5% dimethyl sulfoxide (DMSO) held on ice was used to retain individual leaf explants that were taken from the seedlings. A razor blade was used to carefully slice the leaf tissue explant, and the resulting material was then repeatedly submerged in the ice-cold Sörensen buffer. The suspension of released nuclei was centrifuged at 550 g for 5 min at 4°C after being filtered through a 30-µm disposable filter (Partec, Münster, Germany) to remove the majority of the contaminants.

### 2.3 Isolation of nuclei

A tiny Petri dish with 200 µl of cold, 400-mM Tris-HCl solution at pH 7.5 was filled with leaves (on ice). Under yellow light, the leaves were carefully cut into a “fringe” to release nuclei into the buffer. This technique for isolating nuclei was shown to be the most effective in obtaining low levels of DNA damage in control sample cells. Every slide was previously coated with 1% agaroses with a normal melting point (NMP), dried before being wrapped in a mixture of nuclear suspension and low melting point (LMP) agarose (1% produced with phosphate-buffered saline) at 40°C, and covered with a slip. After at least five min on the ice, the coverslip was removed from the slide. The coverslip was then reinstalled, and 110 µl of 0.5% LMP agarose was gently poured over the slide. After being on the ice for 5 min, the coverslip was slowly removed. Slides used for single-cell gel electrophoresis (SCGE) were subjected for 2 h to the mutagen solutions at 26°C before being rinsed in icy, distilled water thrice for 5 min each time. Slides containing plant cell nuclei were placed inside a horizontal gel electrophoresis container with freshly prepared cold electrophoresis solution (1 mM of EDTA and 300 mM of NaOH, pH > 13) and subjected to 15-min incubation. The electrophoresis protocol was set for 30 min at 300 mA, 16 V, and 4°C. Because they provided minimal levels of DNA damage in control cells and a linear level response for the induction of comets after chemical mutagenic treatment in these cultivars in previous investigations, the electrophoresis conditions utilized in this work were suitable. Ethidium bromide (20 g/ml) was used to stain the gels for 5 min after being neutralized thrice in 400 mM of Tris-HCl (pH 7.5). The gels were stained, submerged in ice-cold distilled water, and examined right away. With the use of a computerized image processing system, a fluorescent microscope equipped with a 546-nm excitation filter and a 590-nm barrier filter was employed to examine 50 cells from each slide at random (Komet Version 3.1, Kinetic Imaging, Liverpool, UK). DNA damage metrics included DNA (TD %), and the tail moment (TM) was measured (Jolanta et al., 2006).

### 2.4 RNA extraction and gene expression

#### 2.4.1 RNA extraction

With the use of plant samples from the *C. aurantifolia* plant, total RNA was isolated (MacRae, 2007). From each treatment, 0.5 g was taken, 500 µl of triazole was added, the sample was thoroughly ground, and 100 µl of chloroform was added. Centrifugation at 10,000 rpm for 5 min was performed. Isopropanol measuring 250 µl was carefully applied after the top layer was removed. The mixture was shaken gently, taking note of the formation of RNA strands, and then placed for 30 min at −20°C in the freezer or until the RNA was collected. The mixture was then subjected to a 5 min of 10,000-rpm centrifugation, after which the filtrate was carefully removed while the small ball formed. Diethyl pyrocarbonate (DEPC) water measuring 500 µl, 25% ethanol, and 75% ethanol were combined and then centrifuged for 5 min. After that, the filtrate was discarded, and the tiny RNA particles were allowed dry before the addition of 50 µl of DEPC water and allowed to dissolve for 15 min in a water bath at 55°C–60°C. With the use of 1% agarose in an agarose gel electrophoresis, the purity of the extracted RNA was confirmed. The agarose was dissolved in 1× TBE buffer to prepare it. With the use of a UV transilluminator (Biometra UV star 15), the isolated RNA was viewed on a 1% agarose gel.

#### 2.4.2 cDNA first-strand synthesis reaction

Following the manufacturer’s instructions, cDNAs were synthesized by incorporating 2 µl of total RNA to a final reaction volume of 20 µl using (Thermo Scientific RevertAid First Strand cDNA Synthesis Kit, Vilnius, Lithuania). The following procedures are part of the cDNA technique. Approximately 1 µl of (dT)18 Primer, RiboLock RNase inhibitor (20 U/µl), Revert Aid RT (200 U/µl), 10 mM of dNTP mix (2 U/µl), 5× Reaction Buffer (4 µl), and nuclease-free water (9 µl) were added. Finally, DEPC-treated water was added, mixed to bring the total volume to 20 µl, and then incubated for 60 min at 42°C. The mixture was then heated for 5 min at 70°C to put an end to the reaction. Therefore, after the cDNAs were obtained, they were chilled for at least 3–5 min.

#### 2.4.3 Polymerase chain reaction

With the High Capacity Access RT-PCR System (Promega, Madison, WI, USA0), reverse transcription was carried out on aliquots of total RNA that had been extracted in accordance with the manufacturer’s guidelines. The chosen primers’ sequences for the genes encoding secondary products RTMYB, pv4CL2RT, TAT, RtHK, RtGPPS, and RtLS (**Table 1**) have been taken into account from earlier research. The master PCR mix was created as follows: 2 µl of cDNA, 0.6 µl of primer, and 4 µl of PCR volumes were added with 12.8 µl of ddH2 and used a negative control, and the master mix total volume was changed to 20 µl. PXE 0.5 thermocycler (Thermo Scientific) was used to carry out semi-quantitative RT-PCR experiments; the cycles were programmed as follows: Stage 1: 94°C, 2–4 min; Stage 2 (40 cycles): 94°C, 30 s, and 61.1°C, 1 min; Stage 3: 68°C, 7 min; Stage 4: hold at 4°C. The results of the SqRT-PCR were observed using standard agarose gel electrophoresis.

**Table 1:** The PCR amplification primers.

Genes	Forward primer 5′–3′	Reverse primer 5′–3′	Terpene biosynthesis pathway genes
RtLS	GGATGTTTCTTCTTGGTCTTCAG	CAGCCTCTTCAAGTACTCTATCT	Linalool synthase
RTMYB	AGTATTGGCAGGAGATCTTCTAC	CCTGAGTCATCTGCAACAATATC	Flavonoids synthase
RtGPPS	CCGGAATCAACAAAGAATACAATAGA	GTAGTACTCATCTGCATGGTTTC	Geranyl diphosphate synthase
Pv4CL2RT	GATATTGTGGGAGAATTGACCAG	GCATGTACAAAGTACACTTTGTGCAG	Hydroxycinnamic acid CoA-ligase
RtHK	ATGGAGTTGCAGAATTCAGCG	CATTTGTTCCAGTACCCAGTATC	Hexokinase
TAT	GTTCTCAGTGGTGGCTCAACTATGT	GGAGTGCCGTTCACAGAAAG	Tyrosine aminotransferase
Actin		GAGGAGCAACCACCTTAATCTTCAT	Housekeeping

#### 2.4.4 Agarose gel electrophoresis

PCR products were analyzed by electrophoresis using a 1.5% gel prepared at 100 V for 90 min, as previously described. The samples were photographed and put on display using UV transelements. The reaction products’ sizes were calculated based on DNA molecular size ranges of 100–1,500 bp. A computer program, GelPro32, was used to quantify the produced bands (Version 4.03).

### 2.5 Statistical analysis

Results are presented as the mean ± standard deviation (SD). All statistical analyses were performed using GraphPad Prism 8 (GraphPad Software, La Jolla, CA, USA). The one-way analysis of variance (ANOVA) was adopted to analyze the data. Significant differences were determined to exist when the *p*-values were less than 0.05.

## 3 Results

### 3.1 Growth parameters of *C. aurantifolia* tissue cultures

**Figure 1:**
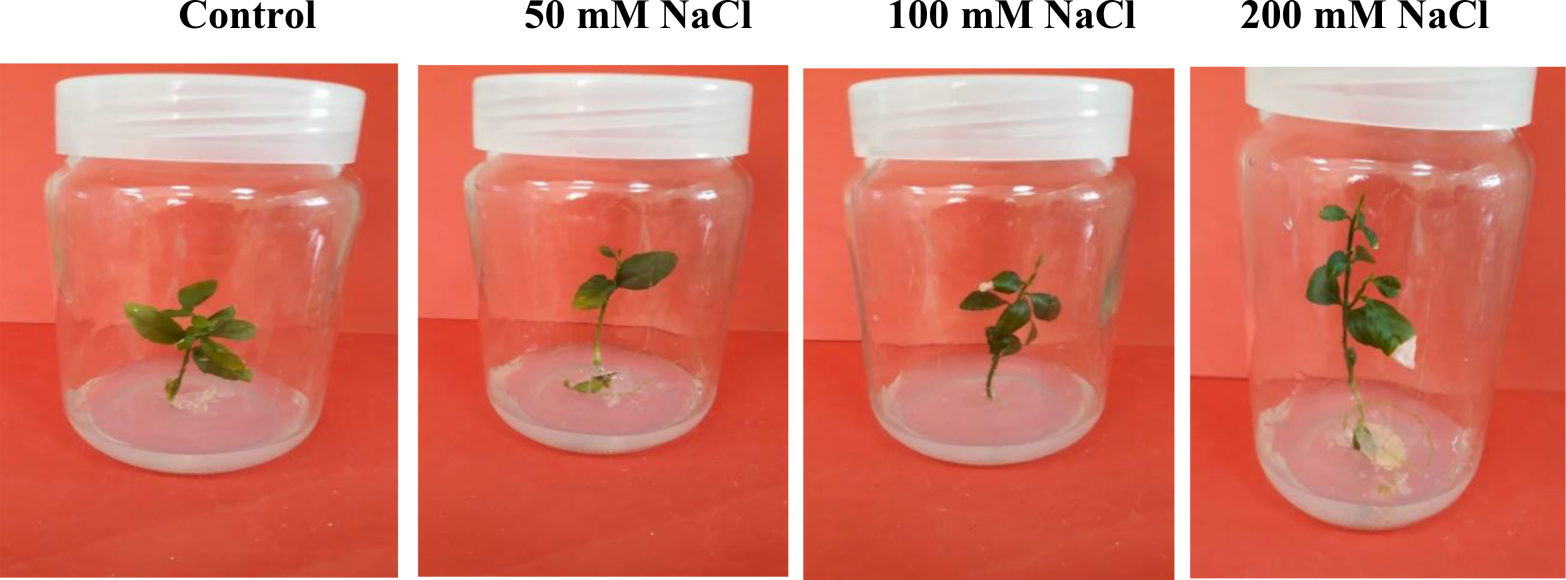
*Citrus aurantifolia* plants cultured in Murashige and Skoog (MS) medium supplemented with 0, 50, 100, and 200 mM of NaCl. After 14 days, growth parameters were assessed.

**Figure 2:**
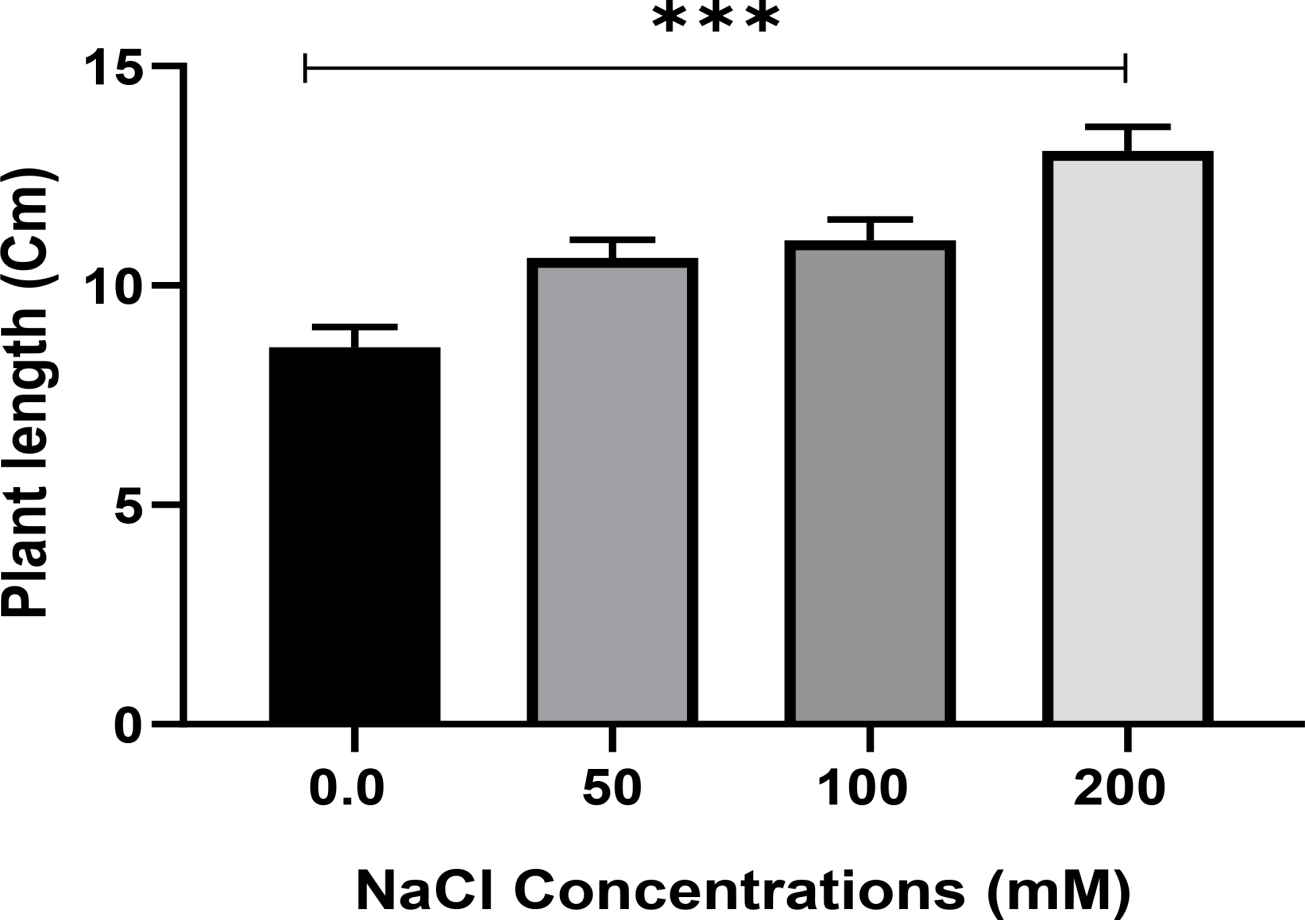
Plant length (cm) of *Citrus aurantifolia* when 10-day-old plants were subjected to varied concentrations of NaCl for further 2 weeks cultivated in full-strength Murashige and Skoog (MS) culture media. *** Significance at *p* < 0.001.

**Figure 3:**
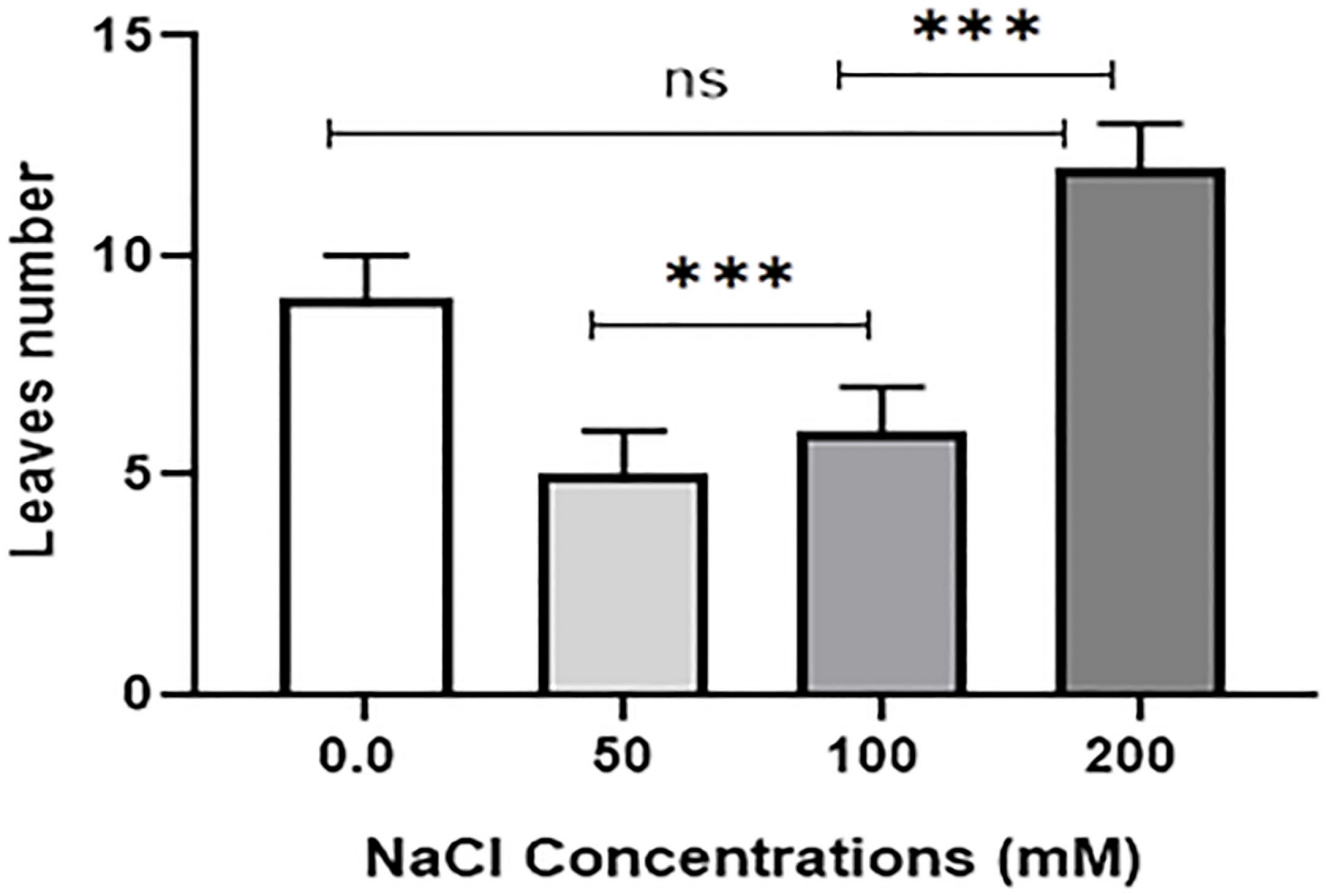
Leaf number of 10-day-old *Citrus aurantifolia* plants subjected to varied concentrations of NaCl for further 2 weeks cultivated in full-strength Murashige and Skoog (MS) culture media. *** Significance at *p* < 0.001; ns, no significant difference.

Salt tolerance of *C. aurantifolia* subjecting under selected levels of salinity (0, 50, 100, and 200 mM of NaCl) was investigated. Our results showed that salinity at 200 mM of NaCl significantly increased plant length and leaf number of *C. aurantifolia* plants with values averaging 13.06 cm and 12, respectively, as shown in **Figures 1**-**3**, whereas the most prominent reduction in growth parameters was observed in plants subjected to MS medium supplemented with 0, 50, and 100 mM of NaCl.

### 3.2 Comet assay (DNA fragmentation-based test)

**Figure 4:**
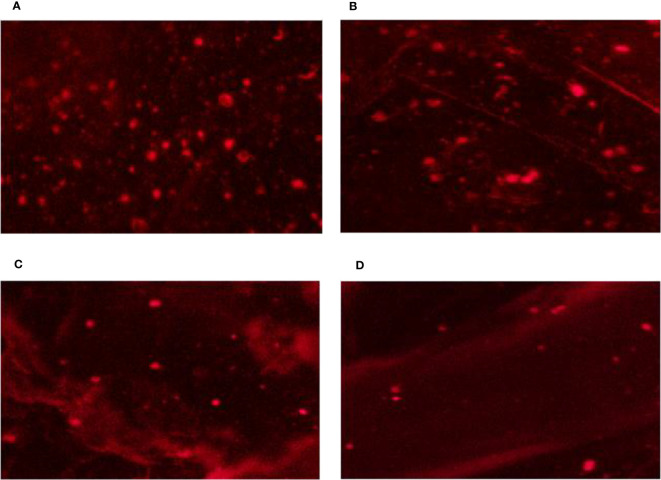
DNA genotoxicity of leaf nuclei from *Citrus aurantifolia* seedling cultured on Murashige and Skoog (MS) medium under different levels of NaCl salinity stress. **(A)** 0.0 mM of NaCl (control). **(B)** 50 mM of NaCl. **(C)** 100 mM of NaCl. **(D)** 200 mM of NaCl.

**Figure 5:**
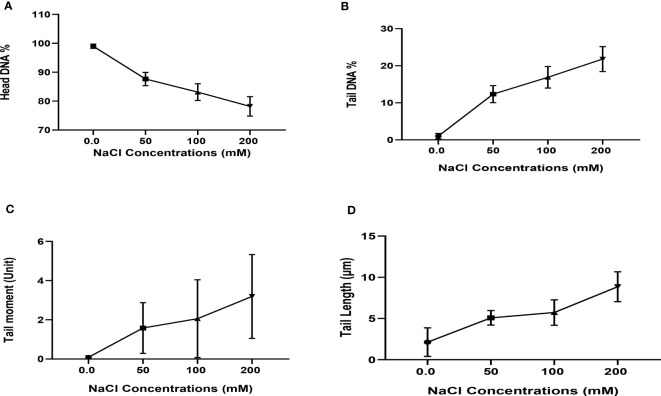
Dose exposure curve of DNA toxicity of nuclei isolated from leaves of *Citrus aurantifolia* culture cells grown on Murashige and Skoog (MS) medium under different levels of NaCl: 0.0, 50, 100, and 200 mM. **(A)** Head DNA%. **(B)** Tail DNA%. **(C)** Tail moment (Unit). **(D)** Tail Length (µm). Means differ significantly (*p* < 0.05).

On MS media supplied with 0.0, 50, 100, and 200 mM of NaCl, DNA damage to the nuclei of each *C. aurantifolia* plant was measured. In this work, several comet assay measurements, including tail length, tail moment, DNA head %, and DNA tail %, were assessed to determine the damage to DNA. The data obtained in **Figures 4**, **5** revealed that dose-scored DNA damage in *C. aurantifolia* plants was recorded at the tail moment 1.58 Unit after 50 mM of NaCl treatment compared with untreated plants (**Figure 5C**). At 200 mM of NaCl level, the expressed damage by % DNA in the tail scored 21.8% (**Figure 5B**) as a highly significant increment compared with untreated plants (Control). The amount of damage scored 16.88% with a significant increment in plants treated with 100 mM of NaCl compared with control plants. The levels of 200 mM of NaCl gave high significant damage by tail length reaching 6.56 µm (**Figure 5D**). Damage expressed by DNA head % was relatively high and reached 78.56%, 83.1%, and 78.18%, when *C. aurantifolia* plants were exposed to 50, 100, and 200 mM of NaCl, respectively (**Figure 5A**). However, DNA damage characterized by tail length and other parameters was not observed in the untreated plants. Comet assay parameters were measured previously as non-significant damage in tail lengths 5.08 and 5.72 µm (**Figure 5D**) when NaCl was added to MS medium at levels of 50 and 100 mM, respectively. The highest reduction in all damage parameters was scored by control plants. Damage appeared to intensify from a level of 200 mM of NaCl (**Figure 5**) in all samples, which led to a significant increase in tail moment, DNA tail %, tail length, and DNA head %.

### 3.3 Gene expression assay

**Figure 6:**
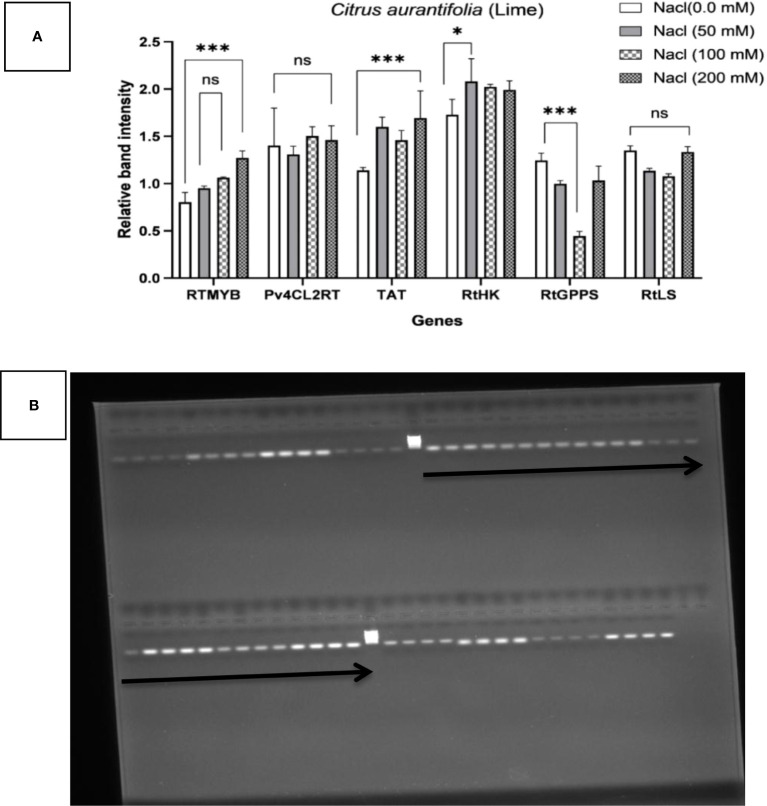
Transcriptional profiling of six genes—RTMYB, pv4CL2RT, TAT, RtHK, RtGPPS, and RtLS—associated with terpene biosynthesis in *Citrus aurantifolia* plant cultures as determined by RT-PCR. Alterations in terpene biosynthesis transcription are connected with the plant exposure to NaCl as a biotic stress in levels of 0.0, 50, 100, and 200. Actin was used as an internal reference for normalization. **(A)** The results are expressed as the mean ± SD (ns, not significant; *p < 0.05, ***p < 0.001, ****p < 0.0001). **(B)** The expression pattern of genes; the top arrow from left to right indicates Ladder, RTMYB, Pv4CL2RT, TAT, and RtHK; the bottom arrow indicates RtGPPS, RtLS, and actin. Each gene has four bands from left to right for 0, 50, 100, and 200 mM, respectively.

The influence of four different NaCl salinity levels on the *in vitro* culture of *C. aurantifolia* stimulated RTMYB, pv4CL2RT, TAT, RtHK, RtGPPS, and RtLS gene expression, demonstrating how alterations in terpene biosynthesis transcription are connected with the plant’s reaction to more NaCl as a biotic stress. Therefore, it was wise to keep an eye on the pathway’s expected precursor number. *C. aurantifolia* plants treated with various levels of NaCl are compared to the control plants and the housekeeping Actin gene, and the recorded alterations connected with these comparisons are shown in semi-quantitative PCR employing six primers of the terpene biosynthesis pathway (**Figure 6**). It has been recorded that flavonoid synthase enzyme (RTMYB), enzyme tyrosine aminotransferase (TAT), and geranyl diphosphate synthase enzyme (RtGPPS) transcript accumulation under salinity stress were provoked in the five treatments if compared with hydroxycinnamic acid CoA-ligase enzyme (Pv4CL2RT), hexokinase synthase enzyme (RtHK), and linalool synthase enzyme (RtLS). *C. aurantifolia* plants considerably reduced the alternate transcript accumulation caused by salt, according to the expression profiles of Pv4CL2RT, RtHK, and RtLS. When *C. aurantifolia* plants were cultivated on MS media supplemented with 200 mM of NaCl, the RTMYB transcript level responded rapidly, as evidenced by the expression profiles (1.26) when compared to control plants (0.8). At 50 and 100 mM of NaCl, salinity-challenged plants showed increased RTMYB expression of 0.95 and 1.06, respectively. When the TAT gene’s transcript accumulation was seen in salt conditions, the covered plants with 200 mM of NaCl scored a significantly higher transcript amount (1.69), compared to the control plants, which scored almost 1.14. It is important to note that the TAT transcript quantity of plants (1.5) cultivated on media containing 50 mM of NaCl was comparable to that of plants cultivated on medium containing 100 mM of NaCl. After recovery, TAT transcripts associated with plants grown in MS media at 50 and 100 mM of NaCl did not show any discernible trend when compared to 200 mM of NaCl. The data in **Figure 6** also show the effect of the chosen genes on terpene biosynthesis transcription in *C. aurantifolia* plants subjected to salinity stress with varying concentrations of NaCl. The results showed that RtGPPS transcript content in *C. aurantifolia* plants treated with 50 mM of NaCl was similar to that in the plants treated with 200 mM of NaCl, which recorded 0.1 each, whereas plants cultured on medium with 100 mM of NaCl 0.5 was assigned to the RtGPPS transcript content. Under conditions of salt stress, there was a progressive decrease in the amount of Pv4CL2RT transcript, whereas the amount of RtHK and RtLS transcript content was at its lowest. RtLS, a crucial enzyme in the linalool synthase pathway, was found in high concentrations and with a specific activity in plants treated with 0, 50, 100, and 200 mM of NaCl (**Figure 6**). Data showed a substantial increase of 1.4 in RtLS transcripts in the control plants when compared to plants treated with salinity stress at 100 and 200 mM of NaCl; in contrast to the previously described treatments, relative band intensity increased significantly when *C. aurantifolia* plants were cultivated in 50 mM of NaCl with value of 2.08, but not significantly when compared to 200 mM of NaCl (1.9). The results clearly indicated that the implicated gene in hexokinase biosynthesis (RtHK) in *C. aurantifolia* plants was affected by NaCl salinity; however, this transcription increase was not substantial at the various salinity concentrations. Similarly, as shown in **Figure 6**, the expression patterns of the genes Pv4CL2RT, RtLS, and RtHK were discovered under salinity stress, and transcription was found to dramatically decrease following salt exposure.

## 4 Discussion

Poplar development is clearly inhibited by salt stress, according to some findings (Ruigang et al., 2008). In this work, the 200 mM of NaCl-stress treatment considerably increased the plant length and leaf numbers of *C. aurantifolia* as a growth parameter when compared to the control. Similar to our findings, the plant height, ground diameter, and crown breadth of *Populus talassica* and *Populus euphratica* were all considerable growth parameters raised by the 200 mmol/L NaCl-stress treatment (Ying et al., 2022). The leaves operated as a salt-elimination mechanism by receiving significant amounts of NaCl from the trees (Chen et al., 1997). Several halophytes exhibit the presence of modified trichomes that are epidermal in origin and are specialized salt secretory structures in their leaves (Shabala et al., 2014). The metric that most accurately depicts DNA damage in our results is the proportion of DNA in the tail, which also expresses the total intensity of the comet as a whole and is independent of the length of the tail. These findings agreed with those of Azqueta et al. (2019) and Gajski et al. (2019), who revealed that the most accurate way to explain DNA break frequencies is to represent them as a percentage of tail DNA since the damage caused by the described comet can be clearly recognized. However, according to Møller et al. (2020), many experts continue to prefer the use of tail moments. In reality, assay conditions have a similar impact on the two classifiers. NaCl is a genotoxic stress that causes transgenic rational and somatic changes in recombination rates. DNA damage caused by applying NaCl to plants was discussed. These changes were initially brought about by exposure to Cl^−^ ions (Boyko et al., 2010; Nikolova et al., 2013). Miriam et al. (2023) characterized different DNA-damaged agents and their mode of action, described DNA damage response (DDR) activation and signaling, and summarized DNA repair mechanisms in plants. Abiotic stresses such as cold, heat, drought, or soil salinity are known to promote reactive oxygen species (ROS) formation and accumulation, which results in oxidative stress that can cause DNA damage (Nisa et al., 2019). Salinity-induced ROS-dependent DNA damage and alteration in plants have not been extensively studied (Huh et al., 2002; Demidchik et al., 2010). According to research by Sihi et al. (2022), the main mechanism by which salinity stress causes DNA strand breaks in rice is the release of ROS. NaCl-induced DNA breaks decrease significantly when ROS-specific antioxidants are applied (Zvanarou et al., 2020; Pedroza-Garcia et al., 2022). An earlier study showed that the genotoxicity of the Al element increased with oxidative stress. Comet test was used to explore the mechanisms of Al genotoxicity, emphasizing the part played by cell wall-bound NADHPX in Al genotoxicity caused by oxidative bursts (Achary et al., 2012) and the mechanism by which Ca^2+^ transmits signals (Achary et al., 2013) and the MAP kinases (Panda and Achary, 2014) in DNA damage and cell death due to Al. According to Monteiro et al. (2012), high concentrations of Cd-DNA create adducts that cause protein cross-links and the formation of longer DNA fragments, as well as the failure of DNA repair processes, which account for these obvious outcomes. Through oxidative stress, which the cell cycle plays a significant part in, two studies on *A. cepa* explored lead-induced genotoxicity through DNA damage (Jiang et al., 2014; Kaur et al., 2014). Boyko et al. (2021) demonstrated that the effect of sodium chloride on genome stability was due to the chloride ion. Chloride ion-supplied media resulted in an increase in the frequency of genomic rearrangements, but sodium ions had no influence on the recombination rates (RRs). It is yet unknown exactly how the Cl ion impact affects the balance of the DNA. Na toxicity causes a striking ion homeostasis deficit in plants. According to Hasegawa et al. (2000), salt stress causes a K+ deficit in the cytoplasm, and Cl− is required for genotoxicity. Four treatments of *C. aurantifolia* plants were subjected to the negative effects of NaCl salt stress, which mostly affected the number of secondary products in the plants. In actuality, under abiotic stresses such as NaCl salt, plants grow slowly, and this is seen as proof of the plants’ ability to adapt and remain healthy under salinity stress (Sabir et al., 2012). Our investigation demonstrated that, despite cell damage limiting development and the presence of obvious sources of metabolite precursors, there was a large buildup of terpenoid synthesis when subjected to salt stress. Terpenoid accumulation was mostly caused by the coordination of pathway-specific genes. Accordingly, glandular cells contain phytochemicals that are generated and are crucial for the plant’s defense against biotic and abiotic stressors (Sangwan et al., 2011). With the use of semi-quantitative RT-PCR analysis, the transcription activity for RTMYB, pv4CL2RT, TAT, RtHK, RTGPPS, and RtLS was compared to that of linalool synthase enzyme, which is expressed strongly in *C. aurantifolia* when grown on a medium that contains 50, 100, and 200 mM of NaCl additions (see **Table 1** for PCR primers used in this investigation). Our results corroborated the findings of Lane et al., 2010 that linalool is the primary constituent of *Lavandula angustifolia* essential oil and that expression of RtLS is directly correlated with linalool accumulation in lavender flowers. A terpenoid synthase can always reverse a single molecule into a variety of compounds; thus, this is not extraordinary (Toll et al., 2005; Degenhardt et al., 2009). The hexokinase synthase enzyme (RtHK) gene, which is thought to be an important enzyme, was clearly expressed with the two treatments given to *C. aurantifolia* plants under the influence of varied salinity levels. According to Graham et al. (1994), RtHK has been proposed as a sugar sensor that inhibits higher plants’ glyoxylate cycle-related genes, and it helps with the expression of sugar-inducible and sugar-repressible genes in these plants. RtHK has been linked to both higher and lower eukaryotes being a sensing substance, despite having been identified as the glycolytic enzyme that accelerates the conversion of ATP-dependent hexoses to hexose 6-phosphates (Jyan et al., 1997). The band intensity of the genes RTMYB, TAT, and RTGPPS seemed to be higher as compared to RtLS, RtHK, and pv4CL2RT genes tested under all salinity levels. Regarding plant RtMYB proteins, they underwent a significant and recent amplification approximately 500 million years ago, around the time land plants first appeared, long before monocots and dicots were distinguished from one another (Rabinowicz et al., 1999; Rensing et al., 2008). This explains how most RtMYB family members control features exclusive to plants, initiating the hypothesis that the expansion was brought about by selection for the control of processes linked to land plants’ sessile nature. Phylogenetic investigations that control the development of trichomes in *Arabidopsis* demonstrate that RtMYB proteins have a close link to regulators of the flavonoid pathway (Serna and Martin, 2006). Anthocyanin biosynthesis regulators in *Arabidopsis* may have undergone gene duplication and subsequent divergence events, leading to the development of RtMYB regulators of trichome formation. This suggests neo-functionalization and that there may be several origins for the regulation of trichome formation. The variation in gene expression observed between seedlings cultivated in different amounts of NaCl salinity can also be explained by variable reactions to environmental cues such as light, temperature, and explant type (Winicov and Shirzadegan, 1997). In this context, our results confirmed the data of Xie et al. (2018), who identified a multitude of transcription factors (TFs) including WRKY, NAC, MYB, AP2/ERF, bZIP, GATA, bHLH, ZFP, SPL, CBF, and CAMTA, which are involved in hormone metabolism, salt overly sensitive (SOS), and ROS signaling pathways in citrus roots. Tyrosine is converted to 4-hydroxyphenyllactate in the tyrosine-derived route by the enzyme TAT. It was discovered that when elicited with MJ, hyphal extracts, or other elicitors, the expression of enzymes in the tyrosine-derived pathway was more closely correlated to the biosynthesis of rosmarinic and salvianolic acid B, suggesting that the tyrosine-derived pathway may be the rate-limiting step in the biosynthesis of phenolic acid (Yan et al., 2006; Zhang et al., 2014). The primary important enzyme for monoterpene biosynthesis (RtGPPS) is often found in plastids and is widely recognized as being plastid-confined (Tholl et al., 2004). GPP synthases in plastids produce the precursor of the majority of monoterpenes from dimethylallyl diphosphate and isopentenyl diphosphate (GPPSs). A catalytic large subunit, such as geranylgeranyl diphosphate synthase, connects with a non-catalytic small subunit (GPPS-SSU), which controls the product specificity of heterodimeric GPPSs (Michael et al., 2013), which showed that RtGPPS is a precursor of almost all monoterpenes, and heterodimeric GPPSs drive a reaction of a catalytic large subunit with a non-catalytic small subunit (GPPS-SSU), such as geranylgeranyl diphosphate synthase, and specifically determine its secondary product. The results of our study were also in line with those recorded on other genes by Shirazi et al. (2019), who mentioned that the two squalene synthase (SQS1 and SQS2) genes, which are involved in the biosynthetic pathway of triterpenoids, have different expression rates in licorice plant due to salinity stress conditions.

## 5 Conclusions

The plant comet test appeared to be an appropriate tool in a related investigation to assess DNA damage induced by exposing plants to known dosages of genotoxins, particularly high levels of salt stress (200 mM of NaCl). The relatively steady expression of RtHK and TAT transcripts under salinity as an abiotic stress in *C. aurantifolia* plants was demonstrated by semi-quantitative RT-PCR. The findings supported the hypothesis that RtHK, TAT, and pv4CL2RT may be subject to post-transcriptional regulation, introducing their abundance in plant cells under salinity stress.

## Data availability statement

The original contributions presented in the study are included in the article/supplementary material, further inquiries can be directed to the corresponding authors.

## Author contributions

HD, GA-O, NA, and AN: conceptualization and writing—original draft. HD, AA, HS, and AN: data curation. HD and AN: formal analysis. HD, AA, NA, and SA-Q: investigation. HD, GA-O, NA, and AN: methodology. HD and AN: project administration. HD, NA, DD, ZA, and AN: resources. HD, AA, NA, AA, and AN: validation. HD, HS, NA, SA-Q, DD, and ZA: visualization. HD, MI, and AN: writing—review and editing. All authors contributed to the article and approved the submitted version.
